# Regulated cell death in neutrophils: From apoptosis to NETosis and pyroptosis

**DOI:** 10.1016/j.smim.2023.101849

**Published:** 2023-11

**Authors:** Léonie Dejas, Karin Santoni, Etienne Meunier, Mohamed Lamkanfi

**Affiliations:** a Laboratory of Medical Immunology, Department of Internal Medicine and Pediatrics, Ghent University, Ghent B-9000, Belgium; b Institute of Pharmacology and Structural Biology, University of Toulouse, CNRS, Toulouse 31400, France

**Keywords:** Neutrophil, Apoptosis, Pyroptosis, Netosis, Cell death, Inflammation, Infection

## Abstract

Neutrophils are among the most abundant immune cells, representing about 50%− 70% of all circulating leukocytes in humans. Neutrophils rapidly infiltrate inflamed tissues and play an essential role in host defense against infections. They exert microbicidal activity through a variety of specialized effector mechanisms, including phagocytosis, production of reactive oxygen species, degranulation and release of secretory vesicles containing broad-spectrum antimicrobial factors. In addition to their homeostatic turnover by apoptosis, recent studies have revealed the mechanisms by which neutrophils undergo various forms of regulated cell death. In this review, we will discuss the different modes of regulated cell death that have been described in neutrophils, with a particular emphasis on the current understanding of neutrophil pyroptosis and its role in infections and autoinflammation.

## 1 Introduction – neutrophil production and clearance

Neutrophils are some of the most abundant immune cells, representing about 50%− 70% of all circulating leukocytes in humans [1]. They originate from hematopoietic stem cells (HSCs) in the bone marrow and progress through various stages of differentiation under the control of granulocyte colony stimulating factors (G-CSF) to become fully differentiated mature neutrophils [2], [3]. In addition to their characteristic multi-lobed nucleus, a prototypical feature of these granulocytes is that they harbour specialized (primary, secondary and tertiary) granules and secretory vesicles containing antimicrobial factors with broad activity against invading pathogens [2], [3].

**Fig. 1:**
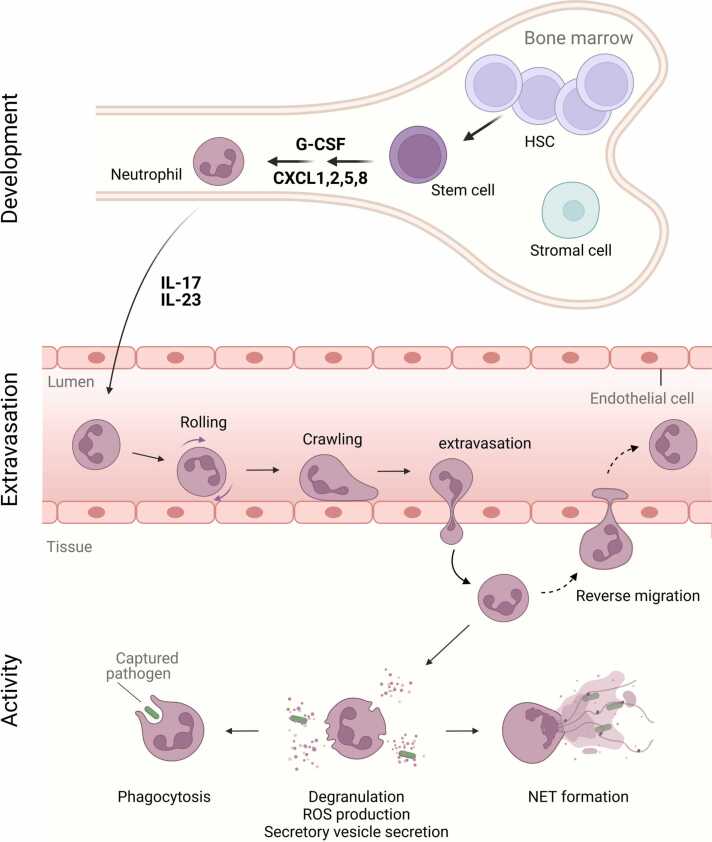
Simplified scheme of neutrophil development, migration and activity. Neutrophils are produced in the bone marrow from hematopoietic stem cells (HSCs) and differentiate into mature neutrophils under the control of granulocyte-colony stimulating factor (G-CSF) and chemokines such as CXCL1, 2, 5 and 8. Mature neutrophils exit the bone marrow to patrol the circulation and migrate into tissues upon detection of microbial or inflammatory signals. At the site of inflammation, neutrophils deploy their arsenal of effector functions including phagocytosis, degranulation, ROS production and neutrophil extracellular trap (NET) formation.

Neutrophil differentiation and release into the circulation is mainly mediated by G-CSF and chemokines such as CXCL1, CXCL2, CXCL5 and CXCL8 that are produced by endothelial cells (Fig. 1). In addition, a complex network of cytokines including interleukin (IL)− 23 and IL-17 produced by professional phagocytes such as macrophages and dendritic cells regulate neutrophil differentiation and egress from the bone marrow under inflammatory conditions [2], [3], [4]. Upon recognition of microbial and/or inflammatory stimuli, neutrophils migrate to the site of inflammation via an adhesion/migration cascade [5]. This process involves several steps starting with the capture, rolling, crawling and eventual extravasation of the neutrophil to the site of inflammation. Once in peripheral tissues, neutrophils are attracted by a concentration gradient of chemoattractants such as CXCL8, IL-1β, leukotriene B_4_ (LTB_4_) and formyl-methionyl-leucyl-phenylalanine (fMLF) to the site of inflammation, where they deploy their effector functions, including phagocytosis, reactive oxygen species (ROS) production, degranulation, secretory vesicle secretion and neutrophil extracellular trap (NET) formation to help cope with the insult [2], [3], [5], [6]. The speed with which they arrive at inflamed tissues and their diverse and broadly effective antimicrobial functions, including proteases and microbicidal peptides, as well as their ability to generate significant amounts of reactive oxygen species (ROS), make neutrophils a cornerstone of host defense against invading pathogens and important modulators of innate and adaptive immune responses [2], [3], [5], [6].

As prototypical short-lived cells, mature neutrophils spend only about one day patrolling the circulation under homeostatic conditions [7]. Maintaining the pool of circulating neutrophils under homeostatic conditions is therefore critically dependent on their constant replenishment from the bone marrow [8], [9]. Activated neutrophils in inflamed tissues can prolong their life cycle under the influence of inflammatory cytokines such as IL-1β and stromal-derived factor 1 (SDF-1) [9], [10], [11]. The end of an acute inflammatory response is accompanied by the release of inflammation-resolving lipid mediators such as lipoxins and resolvins to help limit inflammatory tissue destruction and restore normal tissue homeostasis [12]. During this process, neutrophils undergo apoptosis and phagocytic clearance by resident macrophages in a process called efferocytosis [13]. The clearance of apoptotic neutrophils at the end of acute inflammation also plays an important role in the regulation of neutrophil production in the bone marrow. Efferocytosis of apoptotic neutrophils has been shown to reduce IL-23 production by phagocytes, which acts on HSCs in the bone marrow to inhibit neutrophil production and their release into the circulation [4]. In some cases, neutrophils can re-enter the circulation through a process called “reverse migration”, and return to the bone marrow for clearance (Fig. 1) [14]. Defective neutrophil clearance is thought to contribute to the development of chronic inflammatory diseases.

While neutrophils routinely die by apoptosis under homeostatic conditions [15], recent studies have shown that neutrophils can also undergo alternative modes of regulated cell death including necroptosis [16], pyroptosis [17], [18], [19], [20], [21], [22], [23], [24] and NETosis [25], [26]. Each of these alternative cell death mechanisms has important implications for inflammatory responses and host defense against infections. In the following sections, we will discuss the different modes of regulated cell death described in neutrophils, with particular emphasis on the current understanding of neutrophil pyroptosis and its role in infections and autoinflammation.

## 2 Neutrophil apoptosis and its role in homeostasis

**Fig. 2:**
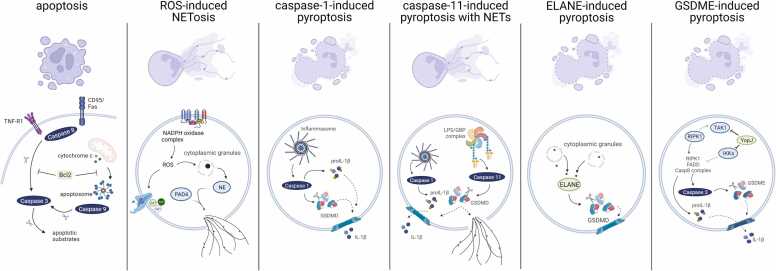
Schematic overview of apoptosis, ROS-induced NETosis and pyroptosis pathways in neutrophils. Apoptosis is a homeostatic and non-inflammatory mode of regulated cell death that is induced by apoptotic caspases and modulated by the expression levels of pro- and anti-apoptotic Bcl-2 family members. In contrast, ROS-induced NETosis and pyroptosis are lytic forms of regulated cell death in which plasma membrane permeabilization leads to leakage of intracellular proteins into the intracellular space. ROS-induced NETosis is associated with expulsion of neutrophils extracellular traps (NETs) following NADPH oxidase-induced ROS production and histone degradation by granule-released serine proteases. Pyroptosis is induced by GSDMD and GSDME pores in the plasma membrane following their cleavage by caspase-1, caspase-11 or ELANE (GSDMD) and caspase-3 (GSDME) in the stimulus-dependent signaling pathways depicted in the figure.

As described above, neutrophils are short-lived cells that are frequently turned over by apoptosis under homeostatic conditions [27]. Apoptosis is an evolutionarily conserved mode of regulated cell death that is generally considered to be a non-inflammatory cell death process. It plays a central role in maintaining organismal homeostasis, and occurs in response to activation of the ‘intrinsic’ or ‘extrinsic’ apoptosis pathways (Fig. 2). These apoptotic pathways initiate an apoptotic caspase cascade that leads to the cleavage of hundreds of apoptotic substrates, thereby giving rise to prototypical apoptotic features such as cell shrinkage, plasma membrane blebbing, apoptotic body formation, chromatin condensation and orderly fragmentation of nuclear DNA [28], [29].

Both the intrinsic and extrinsic apoptosis pathways regulate the survival of neutrophils in the circulation during homeostasis [30], [31], [32]. The intrinsic apoptosis pathway is activated by a variety of intracellular stress factors including DNA damage, hypoxia and metabolic stress. This apoptosis pathway is modulated by the expression levels and activity of pro-apoptotic and anti-apoptotic members of the B-cell lymphoma 2 (Bcl-2) family [29]. Under unstimulated conditions, neutrophils express high levels of pro-apoptotic Bcl-2 proteins that determine their lifespan [30], [31]. Upon activation of the intrinsic apoptosis pathway, the pro-apoptotic Bcl-2 proteins Bcl-2 associated X protein (Bax) and/or Bcl-2 antagonist/killer (Bak) promote permeabilization of the mitochondrial outer membrane (MOMP), resulting in the release of cytochrome c and other mitochondrial proteins into the cytosol [29]. Although cytochrome c expression in neutrophils is low, its release into the cytosol suffices to drive the assembly of the apoptosome, which recruits and activates caspase-9 (Fig. 2). This in turn activates the apoptosis effector caspases 3 and 7 to induce apoptosis [30], [31], [32].

The extrinsic apoptosis pathway, also known as death receptor-mediated apoptosis (Fig. 2), is induced by extracellular ligands of plasma membrane-bound death receptors such as CD95/Fas and various other members of the TNF receptor superfamily (TNF receptor, TRAIL receptor) [28]. As in other cell types, engagement of these death receptors in neutrophils can lead to recruitment of caspases 8 and 10 into a ‘death-inducing signaling complex’ (DISC) that promotes downstream cleavage and activation of caspases 3 and 7 to induce apoptosis [15], [27], [32]. Interestingly, the intrinsic apoptosis pathway can also modulate the kinetics of CD95/Fas-induced apoptosis in neutrophils through the expression levels of the pro- and anti-apoptotic Bcl-2 family members Bid, Bak, Bax, Bcl-2 and Mcl-1 [33]. In addition to these major apoptosis pathways, neutrophils have been shown to release proteases such as cathepsins from primary (azurophilic) granules that cleave and activate caspases 8 and 3 to induce apoptosis [34], [35].

### 2.1 Signaling mechanisms of neutrophil extracellular traps (NETs) and role in ROS-induced NETosis

While apoptosis is a non-lytic and homeostatic mode of cell death, neutrophils can undergo lytic forms of regulated cell death such as ROS-induced NETosis, which is important for host defense against infections [2], [3], [6], [36], [37], [38], [39]. ROS-induced NETosis is an antimicrobial and pro-inflammatory cell death mode in which the NADPH oxidase-dependent oxidative burst plays a central role and that is associated with the assembly of neutrophil extracellular traps (NETs) (Fig. 2). The latter structures were first reported in 1996 and further characterised and renamed to NETs in 2004 [25], [39]. NETs are extracellular web-like structures composed of decondensed DNA that is decorated with cytosolic and granule proteins [2], [6], [39]. Notably, NET formation may accompany several cell death modalities and may result from distinct signaling pathways including calcium flux [26], [40], necroptosis-associated MLKL phosphorylation [16], ROS-induced neutrophil granule protease release [41] and endotoxin-activated caspase-11 [19].

In general, NET formation and release can be divided into three sequential steps (Fig. 2). First, neutrophil activation by pattern-associated molecular patterns (PAMPs) or damage-associated molecular patterns (DAMPs), which bind to several classes of germline-encoded pattern recognition receptors (PRRs), leads to a cascade of downstream signaling pathways that culminate in a concerted increase in cell shape and cell spreading. In a second step, chromatin is decondensed and expanded and translocated DNA in the cytoplasm is associated with cytosolic and granule proteins. Chromatic relaxation and DNA decondensation depends on various cellular proteases that can cleave histones such as neutrophil serine proteases (neutrophil elastase (NE), cathepsin G (CathG), neutrophil proteinase 3 (Pr3)) and caspase-11 [19], [26], [41]. Furthermore, protein arginine deaminase-4 (PAD4) promotes histone citrullination, a post-translational modification that neutralizes the positive charge of arginine to weaken interactions with the negatively charged DNA and facilitate nuclear DNA relaxation and decondensation [40], [42], [43]. Notably, PAD4-independent NET formation has also been reported, suggesting that DNA decondensation may be regulated in a stimulus-dependent manner through redundant signaling mechanisms [3], [26], [44]. In a third and final step, decondensed DNA physically binds neutrophil cytoplasmic granule components such as NE, CathG, Pr3 and myeloperoxidase (MPO) before its extrusion out of the cell through disassembly of the sub-cortical actin network [40], allowing the decorated DNA to be ejected through the permeabilized plasma membrane and into the extracellular environment [2], [3], [6], [26].

NETs have been proposed to exert a bactericidal effect by trapping extracellular microbes, but the extent to which they are critical for neutrophil-mediated restriction of various microbial infections is still debated [3], [36], [38], [39]. In addition to their well-recognized role in trapping invading pathogens, NETs have been implicated in the development of sepsis, autoimmunity, coagulation and cancer [3], [6]. In addition, NETs have been reported to regulate the expression of inflammatory cytokines such as IL-6 and pro-IL-1β in macrophages [45]. Another detrimental role attributed to NET formation is vascular occlusion and the development of thrombosis [46], [47]. Finally, neutrophils have been implicated in promoting cancer development through multiple mechanisms, including the role of NETs in promoting metastatic cells to escape primary tumour sites and spread to different tissues [48], [49], [50].

Excessive ROS-induced NETosis has been shown to increase tissue damage and contribute to the development of severe infections and sepsis [51], [52], [53]. The recent discovery that LDC7559 and its pharmacological analogues potently suppress the NADPH oxidase-dependent oxidative burst that is essential for ROS-induced NETosis may prove valuable to elucidate the mechanisms underlying ROS-induced NETosis in various human and murine disease models [54]. These compounds act by super-agonizing the activity of the glycolytic enzyme phosphofructokinase-1 liver type (PFKL), thereby limiting the cellular availability of NADPH to impair the oxidative capacity of neutrophils [54]. In addition, these agents suggest that hyperactivation of PFKL could potentially be exploited clinically to interfere with the detrimental impact of ROS-induced NETosis in cancer and other non-communicable diseases, while balancing its beneficial effects in antimicrobial host defense.

### 2.2 Canonical inflammasomes and their role in neutrophil IL-1ß secretion

Pyroptosis as a lytic pro-inflammatory mode of regulated cell death was first coined in 2001 [55]. It was initially defined as a caspase 1-dependent lytic cell death mode of infected macrophages [56]. With the discovery of caspase-11-induced pyroptosis in 2011 [57], and the role of gasdermin D (GSDMD)-induced plasma membrane permeabilization in pyroptotic cells [58], [59], pyroptosis was redefined as gasdermin-dependent lytic cell death [60]. Pyroptosis plays an important role in innate immunity against intracellular pathogens by depriving them of their replicative niche and by promoting extracellular release of DAMPs and secretion of the inflammatory cytokines IL-1ß and IL-18 [61], [62].

In macrophages, it is now well-established that pyroptosis can be induced by both canonical inflammasomes (multi-protein complexes that recruit and activate caspase-1) and by caspase-11 (and its human orthologous caspases 4 and 5) in the so-called ‘non-canonical inflammasome’ pathway [28], [56], [57], [59]. At their core, canonical inflammasomes are composed of an intracellular PRR, the bipartite adaptor protein Apoptosis-associated speck-like protein containing a CARD (ASC), and the cysteine protease caspase-1 [28]. Once active, caspase-1 cleaves off a pro-peptide to produce the mature pro-inflammatory cytokines IL-1ß and IL-18. In parallel, caspase-1 cleaves the cytosolic protein GSDMD at the linker region separating its cytotoxic amino-terminal domain from the carboxy-terminal regulatory domain [58], [59]. The released GSDMD amino-terminal domain is then inserted into the plasma membrane, where it oligomerizes into large GSDMD pores. These pores serve as a channel for the secretion of mature IL-1ß and IL-18 [63]. Ionic flux through the GSDMD pores also causes cell swelling, which ultimately leads to pyroptotic cell lysis and the concomitant release of intracellular DAMPs such as HMGB1 [28], [58], [59], [64], [65], [66], [67], [68], [69]. Cell lysis and the release of HMGB1 and other soluble DAMPs from pyroptotic cells has long been thought to be a passive process caused by an unsustainable build-up of osmotic pressure. The recent discovery that the plasma membrane-bound protein Nerve Injury-induced Protein 1 (NINJ1) is required for pyroptotic cell lysis and DAMP release has challenged this notion [70]. A recently reported NINJ1-neutralizing monoclonal antibody that hindered the formation of NINJ1 polymers and effectively reduced liver injury in mouse models of acute hepatitis highlights the potential therapeutic value of neutralizing NINJ1 activity [71]. It is now clear that NINJ1 polymerizes in a fence-like array downstream of GSDMD pore assembly in macrophages [72]. These NINJ1 filaments act as caps on the edges of the membrane, facilitating scission and ultimately enabling the plasma membrane to rupture. However, further investigation is required to elucidate the role of NINJ1 in regulating lytic cell death in neutrophils and determine the extent to which NINJ1-dependent release of DAMPs from dying neutrophils contributes to the development of inflammatory diseases.

Granulocytes are a major source of IL-1β production in pathological conditions, but the canonical inflammasome repertoire capable of producing IL-1β in granulocytic myeloid cells such as neutrophils has only recently begun to emerge [17], [18], [19], [20], [21], [22], [23], [24], [73], [74], [75], [76], [77], [78]. A major challenge in this regard is that neutrophils are short-lived, surviving only up to 12–24 h once isolated. Fortunately, LPS-priming somewhat overcomes this limitation and makes ex vivo inflammasome analysis more tractable [18]. To date, five canonical inflammasomes have been functionally validated in neutrophils ex vivo: three assembled by PRRs of the nucleotide-binding domain, leucine-rich repeat containing (NLR) family (i.e. NLRP1b, NLRP3 and NAIP5/NLRC4), and two that are formed by the non-NLR proteins AIM2 and Pyrin [17], [18], [79], [80]. As in macrophages, activation of the NLRP3 inflammasome by ATP, nigericin or streptolysin in ex vivo cultured neutrophils promotes caspase-1-dependent maturation of IL-1ß [18], [79], [81]. Ex vivo cultured neutrophils also secrete mature IL-1ß upon activation of the AIM2 inflammasome by *Francisella tularensis*, and in response to activation of the Pyrin inflammasome by *Clostridium difficile* exotoxins TcdA and TcdB [18], [23]. GSDMD clearly mediates IL-1ß secretion from inflammasome-activated neutrophils as IL-1ß release by the NLRP3, NLRC4, AIM2 and Pyrin inflammasomes is blunted in GSDMD-deficient neutrophils [17], [18], [73], [82]. In an in vivo setting, neutrophils have been shown to produce IL-1ß in an NLRP3- or NLRC4-dependent manner in mice that have been infected with *Streptococcus pneumoniae* (*S. pneumoniae*), *Salmonella enterica* serovar Typhimurium (*S*. Typhimurium) or *Pseudomonas aeruginosa* (*P. aeruginosa*), respectively [17], [76], [80]. Unlike macrophages, human and murine neutrophils are not a rich source of IL-18 [80], [83].

## 3 Canonical inflammasomes and their role in neutrophil pyroptosis

The role of caspase-1-dependent GSDMD cleavage in IL-1ß secretion from activated neutrophils is well-established, but neutrophils have long been thought to fully resist caspase-1-mediated pyroptosis [19], [80], [82]. The underlying reason has remained enigmatic given that publicly available transcriptome data and immunoblotting analyses show that neutrophils express high levels of GSDMD, comparable to those detected in macrophages [17], [18], [24]. Furthermore, neutrophil elastase (ELANE) - a neutrophil-specific serine protease that gets released from cytosolic granules - cleaves GSDMD slightly upstream of the conventional caspase cleavage site, and ELANE-induced levels of cleaved GSDMD are sufficient to induce pyroptosis in aging neutrophils and during *Escherichia coli* (*E. coli*) infection [24]. It has been proposed that NLRP3 activation fails to induce pyroptosis in neutrophils because caspase-1-cleaved amino-terminal GSDMD primarily targets azurophilic granules and autophagosomes rather than the plasma membrane in neutrophils [82]. However, it is unclear why caspase-11-mediated cleavage of GSDMD at the conventional caspase cleavage site potently induces neutrophil pyroptosis [19], [23]. Thus, while it is clear that GSDMD expression levels are sufficiently high in neutrophils and that ELANE- and caspase-11-mediated cleavage of GSDMD induce neutrophil pyroptosis, a closer analysis of the mechanisms regulating caspase-1-mediated pyroptosis in neutrophils is warranted.

Three recent reports have addressed this question and demonstrated that neutrophils are capable of caspase-1-driven pyroptosis under certain conditions (Table 1). *Pseudomonas aeruginosa* (*P. aeruginosa*) is an extracellular bacterial pathogen that causes pneumonia and induces intoxicated macrophages to undergo pyroptosis and IL-1ß secretion through the NLRC4 inflammasome. A recent report showed that *P. aeruginosa* infection also promotes NLRC4- and caspase-1-mediated GSDMD cleavage in neutrophils [17]. Importantly, infection of neutrophils with engineered mutant strains of *P. aeruginosa* in which ExoS has been inactivated induced significant levels of pyroptosis associated with IL-1β release in wildtype, but not in caspase-1-deficient neutrophils. This suggests that ExoS and ExoU expression suppresses an underlying caspase-1-dependent neutrophil pyroptosis pathway in infected neutrophils. Furthermore, *P*. *aeruginosa* strains expressing the extremely lytic phospholipase exotoxin ExoU induced lysis of infected neutrophils independent of inflammasomes, whereas isogenic deletion of ExoU again unmasked an underlying NLRC4- and GSDMD-dependent pyroptosis program in neutrophils [17]. Separately, activation of the NLRC4 inflammasome in *S.* Typhimurium-infected bone marrow neutrophils has also been shown to induce caspase-1- and GSDMD-dependent IL-1ß cleavage and pyroptosis [18]. FlaTox, a chimeric protein that enters the cytosol via the endolysosomal compartment and activates caspase-1 through the NAIP5/NLRC4 inflammasome, also induces caspase-1-mediated pyroptosis with concomitant IL-1ß secretion (Fig. 2). Similar findings have been reported for *Clostridium difficile* toxin A (TcdA), an enterotoxin that is sensed by the Pyrin inflammasome when it enters the cytosol via the endosomal pathway [18]. However, it is noteworthy that pyroptosis levels induced by these toxins are significantly less pronounced in neutrophils than in macrophages [18]. It is likely that the delivery route by which inflammasome-activating toxins enter the cytosol influences the potency of caspase-1-mediated pyroptosis in neutrophils as electroporation of *Clostridium difficile* toxin B (TcdB) - which delivers TcdB directly into the cytosol – was effective in inducing caspase-1- and GSDMD-dependent pyroptosis in neutrophils [23]. Conversely, cytosolic delivery of TcdB via the endosomal pathway triggered robust caspase-1-dependent IL-1β release and GSDMD cleavage without inducing significant levels of neutrophil pyroptosis [23]. Future studies should investigate the mechanisms involved in modulating the cell death response as a function of how TcdB accesses the cytosol.

**Table 1:** Overview of inflammasome-associated pyroptosis mechanisms in neutrophils.

**Mechanism**	**Stimulus**	**Cell type**	**Experimental protocol**	**Cell death pathway (nomenclature as defined in this review)**	**References**
NLRP3	nigericin, ATP	human and murine neutrophils	LPS (500 ng/ml) for 3 h + nigericin (10 µM) or ATP (3 mM for murine; 4 mM for human) for 45 min	No cell death	[100**]**
	nigericin	murine neutrophils	LPS (100 ng/ml) for 4 h + nigericin (5 µM) for 1–5 h	Cell death (not pyroptosis)	[19]
	nigericin, ATP	murine CD11b+Ly6C+Ly6G+ neutrophils	LPS (250 ng/ml) for 2 h + nigericin (6.7 μM) or ATP (5 mM)	caspase-1/GSDMD-mediated pyroptosis	[18]
NLRC4	*Salmonella* Typhimurium	murine neutrophils	LPS (100 ng/ml) for 4 h + *S*. Typhimurium (SL1344) infection (MOI 0.1–25) for 1–5 h	No cell death	[80]
	*Salmonella* Typhimurium	murine neutrophils	LPS (100 ng/ml) for 4 h + *S*. Typhimurium infection SL1344 (MOI 25) for 1–5 h + gentamycin (100 μg/ml) 25 min after infection	Cell death (not pyroptosis)	[19]
	FlaTox	murine CD11b+Ly6C+Ly6G+ neutrophils	LPS (250 ng/ml) for 2 h + FlaTox [PA (2.5 µg/ml) + LFn-FlaA (2.5 µg/ml)]	caspase-1/GSDMD -mediated pyroptosis	[18]
	*Salmonella* Typhimurium	murine CD11b+Ly6C+Ly6G+ neutrophils	*S*. Typhimurium infection (MOI 25) for 1 h + gentamycin (10 μg/ml)	caspase-1/GSDMD -mediated pyroptosis	[18]
	*Salmonella* Typhimurium inner rod protein PrgJ	murine neutrophils	LPS + IFN-ɣ (100 ng/ml) for 3 h + electroporated PrgJ (4 µg/ml) for 2 h + cytochalasin D (10 µ/ml) 30 min before stimulation	caspase-1/GSDMD -mediated pyroptosis	[23]
	*Pseudomonas aeruginosa*	human and murine neutrophils	*P. aeruginosa* infection (MOI 5 for human; MOI 10 (PAO1 strains) or MOI 2 (PP34 strains) for mouse) for 3 h	caspase-1/GSDMD -mediated pyroptosis without NET formation	[17]
NLRP1b	*Bacillus anthracis* lethal toxin (LeTx)	B6^NLRP1b+^ murine CD11b+Ly6C+Ly6G+ neutrophils	LPS (250 ng/ml) for 2 h + LeTx [PA (2.5 µg/ ml) + LF (2.5 µg/ml)]	caspase-1/11-mediated pyroptosis	[18]
Pyrin	*Clostridium difficile* toxin A	murine CD11b+Ly6C+Ly6G+ neutrophils	LPS (250 ng/ml) for 2 h + TcdA (5 µg/ml)	caspase-1/GSDMD -mediated pyroptosis	[18]
	*Yersinia pseudotuberculosis* ∆*yopM*	murine neutrophils	LPS + IFN-ɣ (100 ng/ml) for 3 h or (50 ng/ml) for 16 h + Y. *pseudotuberculosis* ∆yopM infection (MOI 30) for 3 h + gentamycin (100 μg/ml) 1 h after infection	caspase-1/GSDMD -mediated pyroptosis with NET formation	[23]
	*Clostridium difficile* toxin B	murine neutrophils	LPS + IFN-ɣ (100 ng/ml) for 3 h + electroporated TcdB (10 µg/ml) for 5 h + cytochalasin D (10 µ/ml) 30 min before stimulation	caspase-1/GSDMD -mediated pyroptosis	[23]
Caspase-1/4	*Citrobacter rodentium*	human neutrophils	Pam3CSK4 (1 µg/ml) for 2 h + *C. rodentium* infection (MOI 25) for 30 min + gentamicin (100 µg/ml) 25 min after infection	caspase 1/4 –mediated pyroptosis with NET formation	[19]
Caspase-11	*Salmonella* Typhimurium ΔSifA mutant	murine neutrophils	Pam3CSK4 (1 µg/ml) for 4 h + *S*. Typhimurium infection ΔSifA (MOI 50) for 4 h + gentamicin (100 µg/ml) 25 min after infection	caspase-11/GSDMD -mediated pyroptosis with NET formation	[19]
	Cytosolic LPS	murine neutrophils	Pam3CSK4 (1 µg/ml) for 4 h + LPS transfection (10 µg/ml) for 4 h	caspase-11/GSDMD -mediated pyroptosis with NET formation	[19]
	*Burkholderia thailandensis*	murine neutrophils	Pam3CSK4 (1 µg/ml) for 4 h + *B. thailandensis i*nfection (MOI 100) for 5 h + kanamycin (300 µg/ml) 1 h after infection	caspase-11-mediated pyroptosis	[22]
	*Shigella flexneri* ΔOspC3 mutant	murine neutrophils	Pam3CSK4 (1 µg/ml) for 4 h + *S. flexneri* ΔOspC3 infection (MOI 100) for 4 h + gentamicin (100 mg/ml) 1 h after infection	caspase-11-mediated pyroptosis	[23]
RIPK1/caspase-8	*Yersinia pseudotuberculosis*	murine neutrophils	IFN-γ (100 ng/ml) + LPS (100 ng/ml) for 3 h + *Y. pseudotuberculosis* infection (MOI 10) for 4 h + gentamicin (100 µg/ml) 1 h after infection	GSDME-mediated pyroptosis	[20]
ELANE	*Escherichia coli*	murine neutrophils	*E. coli* infection (MOI 5) for 1 h + kanamycin (50 µg/ml)	GSDMD-mediated pyroptosis	[24]

Although caspase-1-induced neutrophil pyroptosis and ROS-induced NETosis are regulated by different cell death pathways, they both involve chromatin relaxation and DNA decondensation. Citrullination of nuclear histone H3 is a post-translational modification known to play a role in this process by neutralizing the attractive forces between the positively charged histones and the negative charge of the DNA backbone [2], [3], [5], [6]. Indeed, histone H3 citrullination has been documented in both caspase-1-induced neutrophil pyroptosis and ROS-induced NETosis [17], [26]. In caspase-1-induced neutrophil pyroptosis, histone citrullination is mediated by the Ca^2+^-dependent enzyme Protein arginin deaminase-4 (PAD4), which is activated when GSDMD pores allow Ca^2+^ to flux across the plasma membrane and posssibly by dissipating intracellular Ca^2+^ stores at the endoplasmic reticulum. An important difference with ROS-induced NETosis is that caspase-1-induced neutrophil pyroptosis confines decondensed DNA to the intracellular environment [17]. This is in marked contrast to the extensive extracellular NETs formed during ROS-induced NETosis (Fig. 2). The more limited exposure of DAMPs such as histones and NETs to the extracellular environment may contribute to the reduced efficacy of neutrophil pyroptosis in combating *P. aeruginosa* infection [17].

Although many questions remain, it is now clear that neutrophils are capable of caspase-1-dependent pyroptosis. This opens up vast new areas of research into the potential role of canonical inflammasome-induced neutrophil pyroptosis in host defense against acute and chronic infections, and in the etiology of non-communicable inflammatory diseases and metabolic diseases such as non-alcoholic fatty liver disease and atherosclerosis.

### 3.1 Caspase-11 in neutrophils promotes pyroptosis and IL-1β secretion

In addition to the canonical inflammasomes discussed above, neutrophil pyroptosis can be induced by caspase-11 (and its human orthologous caspases 4 and 5) in the so-called ‘non-canonical inflammasome’ pathway (Fig. 2). Activation of the latter inflammatory caspases is coordinated by members of the guanylate-binding protein (GBP) family when lipopolysaccharide (LPS) – a core component of the cell wall of Gram-negative bacteria – is detected in the cytoplasm [84], [85]. Once active, caspase-11 proceeds with direct cleavage of GSDMD to induce GSDMD pore formation and pyroptosis [58], [59]. However, unlike caspase-1, caspase-11 does not cleave pro-IL-1β and pro-IL-18 directly. Instead, caspase-11-generated GSDMD plasma membrane pores allow the efflux of K^+^ ions from the cytosol, leading to activation of the NLRP3 inflammasome and secretion of mature IL-1β and IL-18 [86]. Contrastingly, the human caspase-11 ortholog caspase-4 does cleave pro-IL-18 [87]. Caspase-4 was recently also shown to directly cleave pro-IL-1ß in LPS-transfected human primary macrophages and immortalized epithelial cell lines [88], whereas in THP-1 cells the NLRP3 inflammasome is required downstream of caspase-4 for efficient IL-1β maturation and secretion in response to cytoplasmic LPS [89], [90]. Future research should investigate the underlying mechanisms that dictate whether caspase-4 activation primarily induces direct or NLRP3 inflammasome-dependent cleavage of proIL-1ß in human cells.

In neutrophils, cytosolic LPS-induced regulated cell death has sometimes been defined as NETosis. Although this term has been phenotypically defined as neutrophil cell death that is associated with extracellular NETs, we argue that neutrophil pyroptosis is a better term for LPS-induced neutrophil cytotoxicity given the mechanistic requirement for caspase-11 and GSDMD. Indeed, infection of neutrophils with a mutant strain of *S*. Typhimurium unable to activate the NLRC4 inflammasome (Δ*sifa* mutant) induces caspase-11-mediated neutrophil pyroptosis [19]. As in other myeloid cell types, LPS transfection or infection with the Gram-negative bacterial pathogen *Citrobacter rodentium* induces caspase-11 activation, GSDMD cleavage and IL-1β secretion in human and murine neutrophils [19]. Under these conditions, caspase-11 and GSDMD were also required in neutrophils for nuclear membrane permeabilization, histone cleavage, DNA decondensation and expulsion of expanded DNA as NETs in the extracellular environment [19]. Caspase-11 activation and NET assembly play a critical role in anti-bacterial host defense as mice deficient in caspase-11 had increased bacterial counts. In other work, neutrophils infected with a mutant strain of *Shigella flexneri* lacking a key type 3 secretion system (T3SS) virulence factor (ΔOspC3 mutant) also activated caspase-11 and GSDMD to induce pyroptosis, which again proved important in limiting infection [23]. Similar findings have been reported for infection with wildtype *Burkholderia thailandensis* [22]. Thus, activation of the non-canonical inflammasome and induction of pyroptosis in neutrophils appear to be critical for controlling bacterial infections.

### 3.2 Caspase-8-dependent GSDMD and GSDME activation in *Yersinia*-induced neutrophil pyroptosis

As discussed above, GSDMD is cleaved by caspase-1 during canonical inflammasome-induced neutrophil pyroptosis [17], [18], and by caspase-11 in the context of non-canonical inflammasome-driven neutrophil pyroptosis [19], [22]. Furthermore, leakage of the neutrophil-specific serine protease ELANE from cytosolic granules was shown to induce cleavage of GSDMD slightly upstream of the conventional caspase cleavage site, and to trigger pyroptosis in aging neutrophils and in the context of *E. coli* infection [24] **(**Fig. 2**)**.

In addition to GSDMD, the human genome encodes 5 other gasdermin proteins: GSDMA, GSDMB, GSDMC, GSDME and PJVK [91]. Of these, GSDMD and GSDME are most abundant in hematopoietic cells, with GSDMD being widely expressed in immune cells and GSDME expression being more restricted to macrophages and neutrophils. Rodents lack a GSDMB orthologue, and encode three *Gsdma* genes (*Gsdma1–Gsdma3*) and four *Gsdmc* genes (*Gsdmc1-Gsdmc4*) [91].

Compared to GSDMD, relatively little is known about the role of GSDME in neutrophil cell death. The Gram-negative bacterial pathogen *Yersinia pseudotuberculosis* injects the bacterial acetyltransferase YopJ into target cells to suppress MAP kinase and NF-κB-dependent inflammatory signaling. This induces a regulated cell death response that relies on receptor-interacting serine/threonine kinase 1 (RIPK1) kinase activity and caspase-8 protease activity (Fig. 2). A recent report has clarified the cascade of subsequent cell death events in *Yersinia*-infected neutrophils and in the context of YopJ intoxication [20]. GSDMD undergoes caspase-8-mediated cleavage. In parallel, RIPK1 and caspase-8 promote downstream caspase-3-dependent GSDME cleavage [20]. Analysis of GSDMD-deficient neutrophils revealed that GSDMD plays no role in *Yersinia*-induced neutrophil pyroptosis. Instead, cell lysis was highly dependent on GSDME expression (Table 1). Interestingly, GSDME-driven pyroptosis in *Yersinia*-infected neutrophils was associated with IL-1ß secretion, but failed to extrude NETs into the extracellular environment [20]. This is similar to pyroptosis induced in neutrophils infected with ExoS- or ExoU-deficient *P. aeruginosa*, which also accumulates decondensed DNA in the cytoplasm without significant NET release in the extracellular space [17].

## 4 Neutrophil pyroptosis in autoinflammation

Whereas lymphocytes are the major disease drivers in autoimmune diseases, autoinflammatory diseases are acquired or inborn chronic inflammatory disorders in which innate immune cells are the predominant cause of destructive inflammatory pathology [92]. In addition to their abundant recruitment during the recurrent episodes of non-infectious fever that characterize autoinflammatory diseases, recent studies have begun to shed light on the central involvement of neutrophils in the pathogenesis of several autoinflammatory diseases.

Cryopyrin-associated periodic syndrome (CAPS) is caused by gain-of-function mutations in the inflammasome sensor NLRP3 that trigger excessive inflammasome activation. Subsequent overproduction of IL-1β can lead to periodic symptoms that include intermittent fever, skin rash, arthralgia, severe headache, red eyes and vomiting [92]. Early studies in mice showed that systemic or myeloid cell-restricted (Lysosyme M-Cre-driven) expression of the CAPS-associated NLRP3^A350V^ mutation results in a lethal phenotype associated with neutrophilia and excessive release of IL-1ß [93]. More recently, NLRP3^A350V^ inflammasome activation in macrophages (Fcgr1/CD64-Cre-driven) and neutrophils (MRP8/S100A8-Cre-driven) was shown to independently drive the lethal CAPS phenotype in mice [18], [94], [95]. In contrast to wildtype neutrophils, bone marrow neutrophils from mice expressing the NLRP3^A350V^ allele secrete substantial amounts of IL-1β into their culture medium in response to LPS stimulation alone [18]. Interestingly, culture media from LPS-stimulated mutant neutrophils also contained abundant levels of cleaved caspase-1 [18].

Neutrophil inflammasome activation has also been implicated in Familial Mediterranean Fever (FMF), a recessive autoinflammatory disease caused by mutations in the inflammasome sensor Pyrin [92]. Neutrophils are the most abundant circulating leukocytes during inflammatory episodes in FMF patients. In addition, mice homozygous for the FMF-associated MEFV^V726A^ mutation have been shown to have stunted growth, anemia, systemic neutrophilia and serosal inflammation driven by systemic IL-1ß secretion [96], [97]. In vivo GSDMD deletion completely abolished neutrophilia and all signs of autoinflammatory disease in FMF mutant mice, suggesting a central role for pyroptosis in driving disease in this preclinical FMF model [98]. Future studies should determine the extent to which this effect is recapitulated with neutrophil-selective GSDMD deletion.

Patients with gain-of-function mutations in *NLRC4* can present with severe enterocolitis and life-threatening systemic autoinflammatory disease [92]. Neutrophils may represent an important source of pathological inflammasome activation in these patients, leading to constitutive caspase-1-mediated cleavage of IL-1β and GSDMD. Consistent herewith, neutrophil-restricted NLRC4 inflammasome activation was associated with a severe inflammatory disease characterized by systemic neutrophilia and significant neutrophilic infiltration in almost all tissues, weight loss and joint swelling [99]. The disease is suppressed by injections of anti-IL-1 receptor antibodies. Interestingly, a measurable loss of NLRC4-dependent neutrophils, an indirect measure of pyroptosis, was observed in vivo in diseased mice [99]. However, the extent to which neutrophil GSDMD-driven pyroptosis contributes to neutrophil IL-1 secretion requires further analysis.

## 5 Concluding remarks

Neutrophils are highly abundant in the circulation and are the first cells recruited to sites of inflammation. They participate in the host defense against pathogens through phagocytosis, degranulation, ROS production and the formation of NETs. However, when inflammation persists, neutrophils are continuously recruited to the site of inflammation and can exacerbate tissue damage in non-communicable autoinflammatory, autoimmune and metabolic diseases such as systemic lupus erythematosus, rheumatoid arthritis and metabolic liver syndrome and atherosclerosis.

In addition to apoptosis, necroptosis and ROS-induced NETosis, recent work has identified pyroptosis as an additional mode of regulated cell death that can occur in mature neutrophils. Neutrophil pyroptosis can be triggered by several signaling pathways, including the canonical (caspase-1-dependent) and non-canonical (caspase-11-dependent) inflammasomes, death receptors (RIPK1 and caspase-8) and granule serine proteases (ELANE). Furthermore, depending on the upstream pathways either GSDMD or GSDME may act as pyroptosis executioners in activated neutrophils. Moreover, pyroptosis may or may not be associated with expulsion of NETs, depending on the cell context. In line with the consistent approach taken in this review, we strongly advocate the adoption of a mechanistic nomenclature for regulated cell death in neutrophils. This will facilitate an effective differentiation between different cell death modalities and their pathophysiological functions. Indeed, a growing body of evidence points to both beneficial and detrimental roles of neutrophil pyroptosis in infections and autoinflammation. Importantly, sensitive methods for monitoring plasma membrane permeabilization and extracellular DAMP release are preferred to quantify pyroptosis in neutrophils as neutrophil pyroptosis induced by canonical inflammasomes can be less potent in neutrophils compared to macrophages. Future studies should, among other things, clarify why this is the case, and it will be of interest to determine how the delivery route of microbial toxins modulates pyroptosis induction in neutrophils. Whether and how NINJ1 contributes to DAMP release from neutrophils undergoing ROS-induced NETosis, pyroptosis or secondary necrosis should also be informative. A more detailed understanding of the virulence mechanisms deployed by microbial pathogens to counteract neutrophil cell death modes, and the mechanisms by which lytic neutrophil cell death contributes to cancer, autoimmune and metabolic diseases is urgently needed. With continued progress in this field, innovative approaches to pharmacological modulation of neutrophil cell death in human disease may finally be within reach.

## Declaration of Competing Interest

M.L. serves as a consultant for Ventyx Biosciences and Novo Nordisk outside of the submitted work. The other authors declare that they have no conflict of interest.
